# Cryptic Sites in Tau Fibrils Explain the Preferential Binding of the AV-1451 PET Tracer toward Alzheimer’s Tauopathy

**DOI:** 10.1021/acschemneuro.0c00340

**Published:** 2021-06-21

**Authors:** N. Arul Murugan, Agneta Nordberg, Hans Ågren

**Affiliations:** † School of Engineering Sciences in Chemistry, Biotechnology and Health, KTH Royal Institute of Technology, S-106 91 Stockholm, Sweden; ‡ Division of Clinical Geriatrics, Center for Alzheimer Research, Department of Neurobiology, Care Sciences and Society, Karolinska Institutet, S-141 86 Stockholm, Sweden; ¶ Theme Aging, The Aging Brain, Karolinska University Hospital, Huddinge, S-141 86 Stockholm, Sweden; § Department of Physics and Astronomy, Uppsala University, Uppsala SE-75120, Sweden; ∥ College of Chemistry and Chemical Engineering, Henan University, Kaifeng, Henan 475004, P. R. China

**Keywords:** Tau imaging, neurofibrillary tangles, multiscale modeling, Alzheimer’s disease, Pick’s disease, chronic traumatic encephalopathy, QM fragmentation scheme

## Abstract

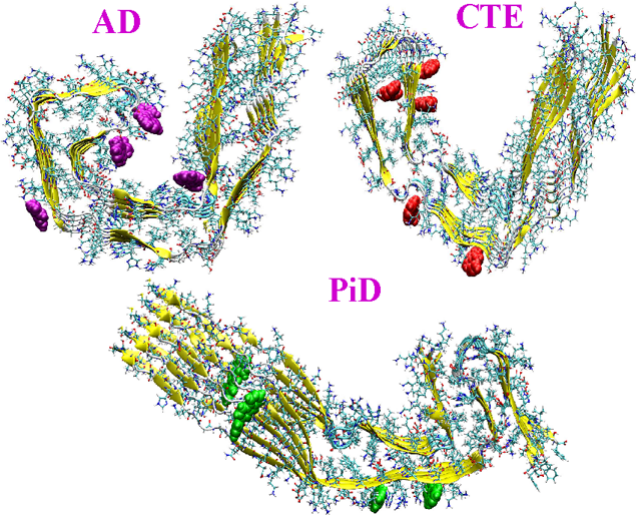

Tauopathies are a subclass of neurodegenerative diseases characterized by an accumulation of microtubule binding tau fibrils in brain regions. Diseases such as Alzheimer’s (AD), chronic traumatic encephalopathy (CTE), Pick’s disease (PiD), and corticobasal degeneration (CBD) belong to this subclass. Development of tracers which can visualize and discriminate between different tauopathies is of clinical importance in the diagnosis of various tauopathies. Currently, several tau tracers are available for in vivo imaging using a positron emission tomography (PET) technique. Among these tracers, PBB3 is reported to bind to various types of tau fibrils with comparable binding affinities. In contrast, tau tracer AV-1451 is reported to bind to specific types of tau fibrils (in particular to AD-associated and CTE) with higher binding affinity and only show nonspecific or weaker binding toward tau fibrils dominant with 3R isoforms (associated with PiD). The tau fibrils associated with different tauopathies can adopt different microstructures with different binding site microenvironments. By using detailed studies of the binding profiles of tau tracers for different types of tau fibrils, it may be possible to design tracers with high selectivity toward a specific tauopathy. The microstructures for the tau fibrils from patients with AD, PiD, and CTE have recently been demonstrated by cryogenic electron microscopy (cryo-EM) measurements allowing structure-based in silico simulations. In the present study, we have performed a multiscale computational study involving molecular docking, molecular dynamics, free energy calculations, and QM fragmentation calculations to understand the binding profiles of tau tracer AV-1451 and its potential use for diagnosis of AD, CTE, and PiD tauopathies. Our computational study reveals that different affinity binding sites exist for AV-1451 in the tau fibrils associated with different tauopathies. The binding affinity of this tracer toward different tau fibrils goes in this order: PiD > AD > CTE. The interaction energies for different tau fibril–tracer complexes using the QM fragmentation scheme also showed the same trend. However, by carrying out molecular dynamics simulations for the AD-derived tau fibrils in organic solvents, we found additional high affinity binding sites for AV-1451. The AV-1451 binding profile in these cryptic sites correctly explains the preferential binding of this tracer toward the AD fibrils when compared with the PiD fibrils. This study clearly demonstrates having a cryo-EM structure is still not sufficient for the structure-based tracer discovery for certain targets, as they may have “potential but hidden” high affinity binding sites, and we need additional strategies to identify them.

## 1 Introduction

Several neurodegenerative diseases are characterized by accumulation of certain biomolecular aggregates in different brain regions at the intraneuronal and extraneuronal compartments.^1^ Various proteins such as the amyloid beta, tau, alpha-synuclein, Huntingtin, and amylin can form amyloid aggregates that are rich in β sheet contents.^2^ Tauopathies are a class of neurodegenerative diseases associated with the accumulation of fibrils of microtubule binding tau proteins.^3,4^ Alzheimer’s disease (AD), Pick’s disease (PiD), chronic traumatic encephalopathy (CTE), and corticobasal degeneration (CBD) present different types of tauopathies with different clinical phenotypes.^5^ The key role of tau protein is to stabilize the microtubule structure that is responsible for the transport of nutrition to the brain region. It is proposed that the hyperphosphorylation of tau proteins leads to aggregate formation, and tau fibrils with straight, twisted, or paired helical filaments are usually formed. They make up for highly insoluble neurofibrillary tangles (NFTs) which lead to a breakdown of the microtubules and to malfunctioning of neurons. It has been a main objective to estimate the population of such NFTs as they can be directly correlated to premorbid cognitive dysfunction^6^ and neuronal loss and disease progress. Many radiolabeled tau tracers have so far been developed and tested in vivo for imaging of regional accumulation of tau in AD and other tauopathies.^7−9^ The first generation tau tracers such as the FDDNP, PBB3, and the THK series compounds AV-1451 and T808 are tracers binding to different tauopathies,^7,9^ while the second generation tracers such as JNJ-311, MK-6240, Ro-948, and PI-2620 seem to exhibit considerably high binding affinity toward tau fibrils in AD.^7,9,10^ While the focus so far has been on AD, only a few tau tracers have been tested for binding affinity toward tau fibrils from different tauopathies like PiD, CTE, CBD, progressive supranuclear palsy (PSP), and argyrophilic grain disease (AgD).^9,11^ These diseases are generally referred to as non-AD tauopathies since they are associated with tau fibril accumulation but not with the co-occurrence of amyloid beta fibrils as in AD.

**Figure 1:**
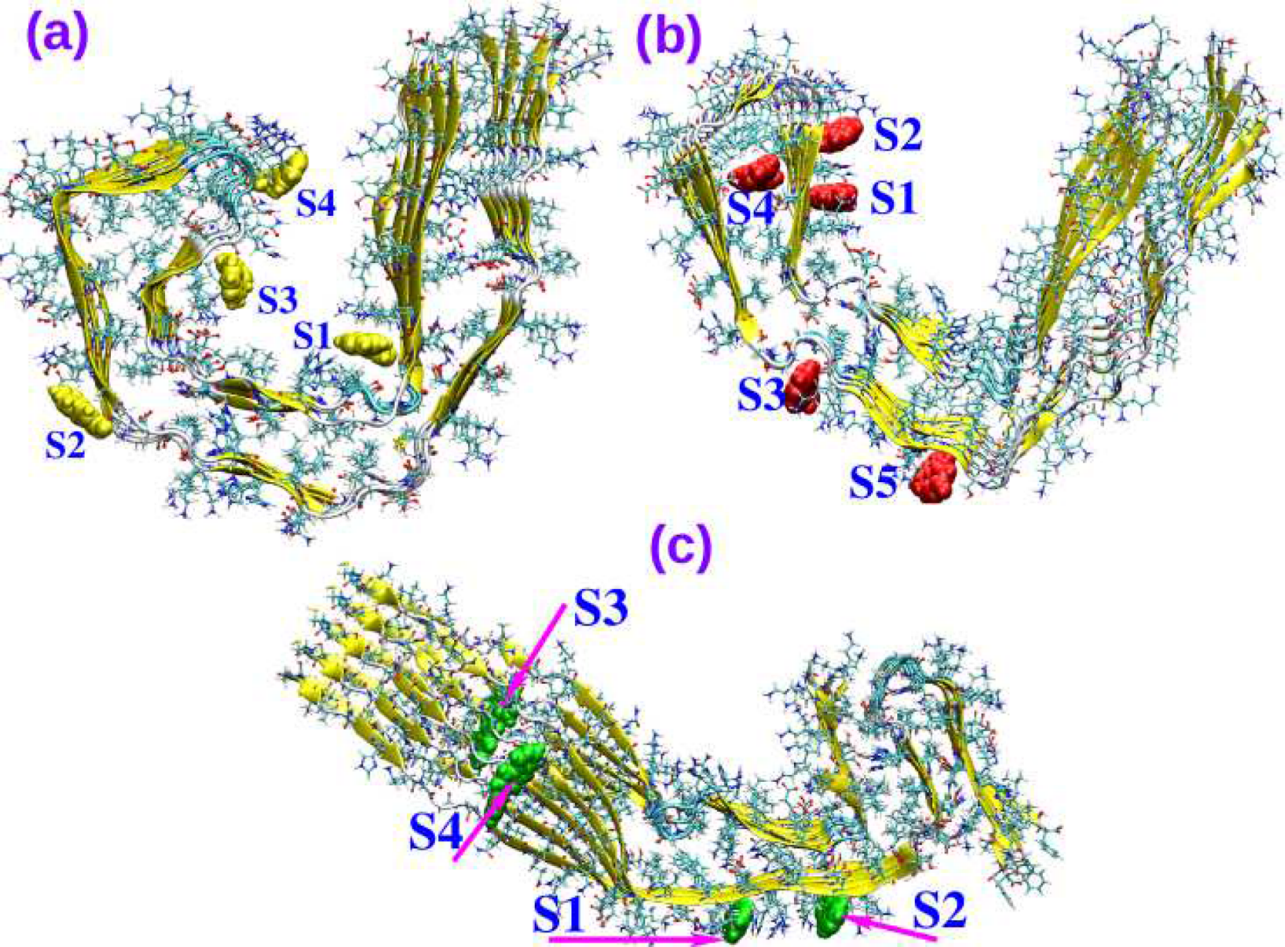
Binding modes of AV-1451 in tau fibrils from (a) AD, (b) CTE, and (c) PiD patients. Various high affinity binding sites for the tracer are shown.

It is known that the variation in the primary structure of the tau protein is correlated to different types of tauopathies.^5^ The tau protein can exist in six isoforms, and the number of amino acid residues in each form can range from 352 to 441. Depending upon the specific isoform, the tau protein can have three (3R) or four repeat (4R) units which bind to microtubules and stabilize their microstructure.^5^ It has been reported that one or other isoforms dominate a specific case of tauopathy. For example in AD, CTE, and Down’s syndrome, the 3R and 4R isoforms dominate, while in Huntington’s disease, PSP, AgD, and CBD are dominated by the 4R isoform.^9^ Pick bodies of Pick’s disease are dominated by 3R isoforms. Due to recent developments in cryogenic electron microscopy (cryo-EM) measurements, the structures for many fibrils have now been solved which were elusive earlier.^10−13^ Thus, the structures for tau fibrils from AD, CTE, and PiD patients have become available recently.^10,11,13^ The first report of a tau fibril structure from an AD brain (will be referred to as AD-tau) was demonstrated by Fitzpatrick et al. in 2017 which showed a double C-type structure (where the outer C filament surrounds the inner filament). The fibril growth occurs in a direction perpendicular to this double C-type structure, and infinitely many fragments are aligned in parallel to make up for the insoluble neurofibrillary tangles (refer to Figure 1a).^10^ Interestingly, the tau fibril structure reported from a CTE brain (will be referred to as CTE-tau) also had a similar structure except that it had a wider opening in the closed region (refer to Figure 1b) and more open space within the closed filaments.^11^ Later we will discuss about the alignment of structures of AD-tau and CTE-tau. However, the tau fibrils from a PiD brain (referred to as PiD-tau) had an altogether different microstructure with a more open region and with additional folds at the C-terminal region (refer to Figure 1c). Moreover, the tau filaments can as well exist with straight, twisted, and paired helical patterns which differ with respect to the interfragments packing. In the straight and twisted cases, the filaments are arranged in a back-to-back fashion, while in the paired-helical case, a base-to-back arrangement occurs.

It is of great clinical interest to develop tau tracers which can bind to a specific type of microstructure of tau fibrils and thereby detect different variants of tauopathy. It might be necessary to develop tau tracers which can light up multiple tauopathies by binding to both tau microstructures with 3R and 4R units and that exist both as straight and paired-helical filaments. In the latter case, the aim is to design a unique tracer to detect all possible tauopathies. Since the tau accumulation in different brain regions has typical distribution patterns, the tauopathies can be subclassed into specific types based on the spatiotemporal distribution of tau fibrils.^14−16^ There are not many tau tracers which have been demonstrated to bind to microstructures of tau fibrils associated with different tauopathies. The tau tracers PBB3 and AV-1451 (also known as flortaucipir or T807) have demonstrated significant binding to non-AD tauopathies.^17−22^ The PBB3 tau tracer was the first tau tracer to detect non-AD tauopathies like FTD, PSP, and CBD.^17,18^ The tracer also was reported to bind in an AD brain to multiple binding sites with high binding affinity.^18^ Autoradiography studies with brain samples from patients with AD, PiD, and PSP have demonstrated that PI-2620 binds to these tauopathies, but no specific binding was reported for the brain slices of nondemented subjects.^23^ Similar studies with AD- and PSP-derived brain homogenates suggested that AV-1451 binds to tau fibrils associated with AD with high specificity but nonspecifically was binding to PSP. The elevated signals for this tracer when compared to controls in the brain regions such as pallidum, midbrain, dentate nucleus of the cerebellum, thalamus, caudate nucleus, and frontal regions can be associated with PSP.^15,19^

In addition to its potency as a diagnostic tracer for AD and PSP tauopathies, AV-1451 also displayed its ability to image CTE effectively.^20^ Football players with a history of repetitive head injuries tend to develop CTE neurodegenerative disease, and a recent study with AV-1451 in living players could reveal increased tau deposition in the brain when compared to normal nonplayers.^24^ Further, it has been reported that AV-1451 also binds to CBD-associated tau fibrils, and its retention in motor cortex, corticospinal tract, and basal ganglia could be correlated to CBD.^15,25,26^ Autoradiography studies with brain tissues from patients with Pick’s disease also showed AV-1451 binding to tau deposits associated with Pick’s disease.^27^ However, it was found that the binding affinity for AD-associated tau fibrils was stronger than for the PiD case.^27^ Another study with post-mortem brain tissues from patients with different tauopathies showed that the tracer binding to PiD tauopathy is moderate, and that the tracer uptake can be used to delineate AD and PiD cases from other tauopathies.^28^ All these experimental studies reveal that AV-1451 binds to both 3R+4R tau fibrils (as in AD) as well as to either 4R tau (as in CBD) or 3R tau fibrils (as in PiD),^16,27^ but the binding of AV-1451 to non-AD-associated tau fibrils has been reported to be either nonspecific or weak when compared to AD.^16,27^ Overall, AV-1451 appears promising to image a wide range of non-AD tauopathies such as PSP, CTE, and CBD in addition to AD. It is worth recalling that this tracer is also reported to bind to a number of off-targets such as monoamine oxidase-A, monoamine oxidase-B (MAO-B), and neuromelanin-containing cells from the substantia nigra.^29,30^ We have recently studied the off-target binding of AV-1451 to the MAO-B target using in vivo and computational studies.^31^

**Figure 2:**
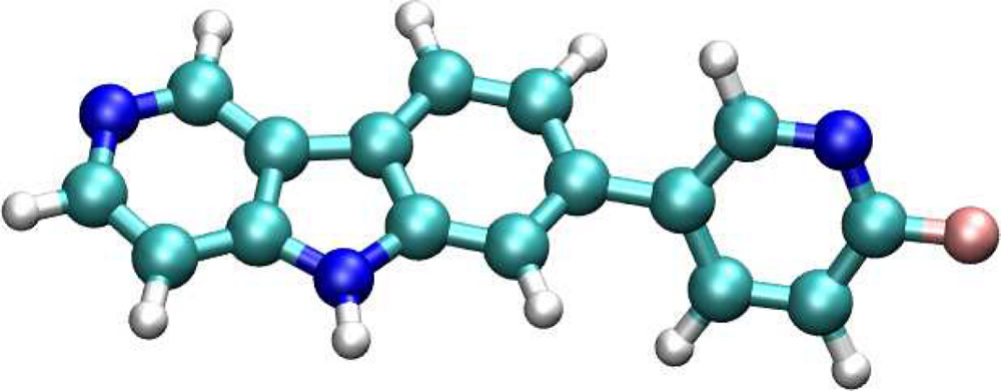
Molecular structure of tau tracer AV-1451.

In this work, we intend to address the mechanism behind the binding affinity of AV-1451 (refer to Figure 2 for its chemical structure) toward various tau microstructures associated with different tauopathies. Until now the tau fibril structures from the patients of AD, CTE, and PiD are only available, and so we considered these targets.^10,11,13^ We carried out blind molecular docking to find out various possible binding sites for AV-1451 in three different tau fibrils. Molecular dynamics simulations in the isothermal–isobaric ensemble for the AV-1451 bound to different binding sites of tau fibrils were carried out to address the stability of fibril–tracer complexes and to establish the equilibrium structures of tracer-bound tau fibrils. Finally free energy calculations based on the MM-GBSA approach^32^ were carried out to estimate the relative binding affinity of AV-1451 in different binding sites of different tau fibrils. To validate the force-field-based binding affinity, we also carried out calculations using the QM fragmentation scheme^33−35^ and computed the total interaction energy between the fibril and tracer at the M06-2X/6-31+G* level of theory which is known to describe the stability of intermolecular complexes having even weaker interactions.^36^

## 2 Results and Discussion

### 2.1 Binding Profile of the AV-1451 Tracer toward Tau Fibrils Derived from AD, CTE, and PiD Cases

**Figure 3:**
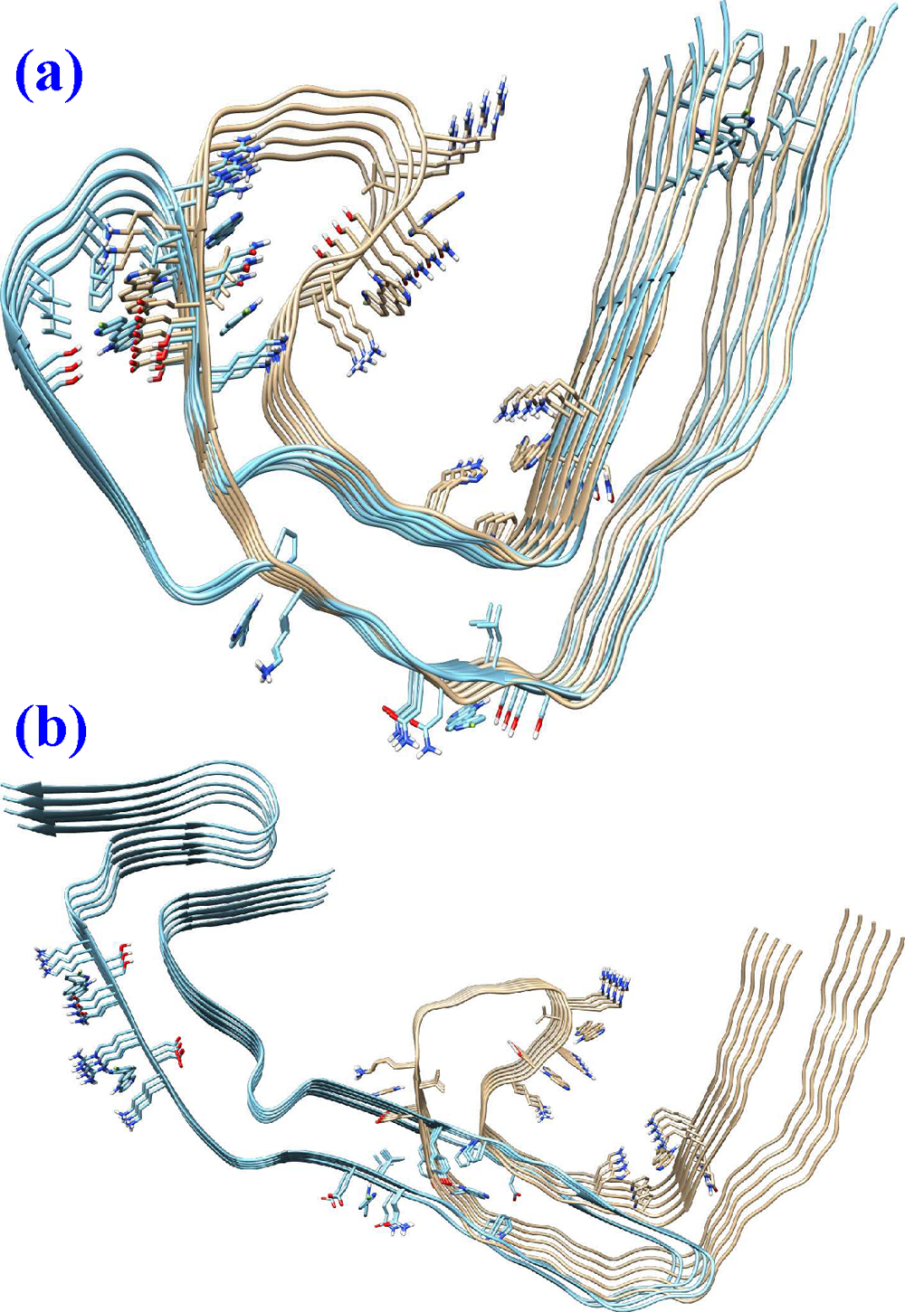
(a) Overlap of microstructures of tau fibrils from AD and CTE patients and (b) overlap of microstructures of tau fibrils from AD and PiD patients.

The molecular docking studies show multiple binding sites available for AV-1451 in different tau fibrils (refer to Figure 1a–c). The number of binding sites is comparable for the tau fibrils from AD and Pick’s disease patients. In the case of tau fibrils associated with CTE (i.e., CTE-tau), there is an additional core binding site for AV-1451. From the cryo-EM measurements of CTE-tau fibrils and analysis, it has been suggested that a steroid-like hydrophobic molecule is bound to this site.^11^ It is interesting to note that this specific site in the CTE-tau fibril also is targeted by the tracer. Given that the primary structure is the same for both AD-tau and CTE-tau fibrils, the difference seen in the location of the binding sites is quite intriguing. The binding of AV-1451 in sites 3 and 4 (refer to Figure 1a) is the only common feature for both of these fibrils. To further understand the reason for AV-1451 choosing different sites for binding, we merged the two fibrils as shown in Figure 3a. Even though the two microstructures largely show similarities (major overlap of the tertiary structure is shown in Figure 3a), the curvatures seen in certain parts seem to influence the ligand binding in those regions. For example, site 1 binding of the AD-tau fibril is missing in the case of CTE-tau which has to be attributed to the increased curvature in the latter case. Similarly, the increased curvature in the region around the residues GLY61, GLY62, GLY63, ASN64, and LYS65 in the case of CTE-tau appears to be responsible for AV-1451 not binding to this site. As we mentioned above, the binding of AV-1451 to an additional core site in the CTE-tau fibril should be attributed to the more open region and availability of a cavity with a hydrophobic microenvironment. A careful analysis of this site suggests that the hydrophobic residues LEU40, PHE42, and VAL46 are contributing to the core site with favorable cavity volume to drive AV-1451 binding to this site. In addition to two interlinked aromatic moieties, which facilitate favorable interaction with the hydrophobic core, AV-1451 also contains NH and pyridyl groups which can interact with polar residues. As can be seen, the SER38 interacts favorably with the polar NH group of AV-1451 further contributing to the stability of the fibril–tracer complex. It is notable that this particular site constitutes to the major difference between the AD-tau and CTE-tau fibrils. So, if ligands with binding specificity to this site can be designed, it could probably be used to detect CTE related tauopathy selectively. There is also another possibility that this site becomes available in AD-tau in the vicinity of the ligand through an induced fit-mechanism, and we will explore this possibility in the next section.

Turning to the AV-1451 binding to tau fibrils from Pick’s disease patients, we find that first of all that the microstructure of PiD-tau is quite different, and the merging of its structure with the AD-tau fibril (refer to Figure 3b) does not show any resemblance. It is quite remarkable that an excess of one β sheet repeat unit contributed from the residues 1–72 of PiD-tau yields very different folds and binding sites for AV-1451. As can be seen, there are three surface binding sites and a single core binding site. The core binding site is made of residues such as TYR26, PRO28, VAL29, PRO48, GLY49, GLY50, and GLY51 which are hydrophobic residues except TYR26 which satisfies the interaction with the polar group of AV-1451. Overall, in both cases of CTE-tau and PiD-tau, the interaction between AV-1451 and fibrils in the core site is dominantly driven by hydrophobic interactions and also to some extent through the intermolecular hydrogen bonding interaction of SER44 (in the case of CTE-tau) and TYR26 (in the case of PiD-tau). We will discuss below about the relative binding affinities of AV-1451 in different binding sites. It will be interesting to see whether the core sites are those ones associated with higher binding affinity. It is here notable that CTE-tau and PiD-tau have a single binding site where there is no access to a water environment except along the fibril growth axis (refer to site 5 of CTE-tau and site 3 of PiD-tau). These fibrils are generally referred to as steric-zippers^37^ due to their tightly packed nature which arises because of the complementarity between the residues along the filament and multiple intermolecular hydrogen bonding interactions between the filaments. This gives very strong stability of these structures and the least free volume which probably is the reason behind that the enzyme-mediated clearance of these fibrils is not working efficiently. So, it is understandable that there are not many core sites available in these fibrils which would have given leads to the fibril clearing degrading enzymes to work on. Moreover, as we have seen, the core sites’ microenvironments are unique to each tau fibril, and so targeting such sites with tracers can lead to development of tauopathy-specific tracers. A careful analysis of interaction between the tracers and binding site microenvironment might pave a way to succeed in this.

The binding free energies computed from the MM-GBSA approach for AV-1451 in all major high affinity binding sites are provided in Table 1. The binding free energies are proportional to the binding affinities. The lower the binding free energies, the larger the binding affinities are. In addition, the binding free energies are related to experimental inhibition constants by the relationship as given below:
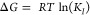
1

**Table 1:** Binding Free Energies of AV-1451 in Different Binding Sites of a Single Filament of Tau Fibrils from Patients with Alzheimer’s Disease, CTE, and Pick’s Disease (in kcal/mol)^a^

site	*ΔE*_*vdw*_	*ΔE*_*elec*_	*ΔG*_*GB*_	*ΔG*_*SA*_	*ΔG*_*binding*_
AV-1451 with AD-tau					
site 1	–39.2	–11.8	24.1	–4.3	–31.2
site 2	–13.0	–0.3	6.2	–1.3	–8.4
site 3	–24.7	–15.1	24.1	–3.0	–18.6
site 4	–26.5	–24.6	35.3	–3.4	–19.3
AV-1451 with CTE-tau					
site 1	–35.3	–7.9	24.7	–4.2	–22.7
site 2	–35.6	–20.9	36.3	–3.8	–24.0
site 3	–27.3	–24.2	34.5	–3.0	–20.1
site 4	–30.0	–14.0	22.6	–4.3	–25.8
site 5	–30.9	–6.8	17.5	–2.9	–23.1
AV-1451 with PiD-tau					
site 1	–27.6	–9.5	21.3	–3.4	–19.2
site 2	–31.5	–12.7	25.9	–3.7	–21.9
site 3	–41.2	–19.0	28.9	–4.6	–35.9
site 4	–19.7	–7.2	16.6	–2.3	–12.5

a The results are obtained from the MM-GBSA approach.

The binding free energies are the sum of electrostatic, van der Waals, and polar and nonpolar solvation energies. The first two terms refer to the interaction between the fibril–tracers in a gas-like environment, while the last two terms account for their interaction in a solvent-like environment and are referred to as the solvation energy. In the case of AD-tau and PiD-tau fibrils, the magnitude of free energies of binding varies significantly depending upon the binding site. In these cases, at least one high affinity binding site (the binding free energy in the range −31 to −36 kcal/mol), sites with moderate binding affinity (the binding free energy in the range −18.0 to −22 kcal/mol), and sites with not so significant binding affinity (with binding free energy in the range −8 to −13 kcal/mol) are seen. In the case of the AD-tau fibril, site 1 is the one associated with high binding affinity, and sites 3 and 4 are associated with moderate binding affinity (refer to Figure 1a and Table 1). Similarly, in the case of PiD-tau, there is a binding site (site 3) with high affinity, and sites 1 and 2 are with moderate binding affinity. It is also worthwhile to mention that, in this case, the core site is the one associated with larger binding affinity when compared to other surface sites. In the case of CTE-tau, almost all the sites have comparable binding affinity with the magnitude of binding free energies in the range −20 to −25.8 kcal/mol. It may be of relevance to compare the binding profile data we get from modeling with experimental data. Unfortunately, detailed experimental data from the autoradiography, fluorescence, and binding assay studies on brain homogenates are available only for the case of AV-1451 with the AD-tau fibril, and for the cases of tau fibrils associated with Pick’s disease and CTE only limited studies are available indicating that the binding of AV-1451 to these fibrils is less strong when compared to the former case.^27^ There are also head-to-head comparisons of PET studies of AV-1451 and tau tracers such as PBB3, THK5351, and THK5117 available which provide information about whether the two tracers target a unique binding site or independent sites within AD-derived tau fibrils.^18,29,38^ These studies revealed that there are at least two different binding sites for AV-1451 in tau fibrils from AD, and we would like to relate this to high affinity and moderate affinity binding sites as we discussed above.

**Figure 4:**
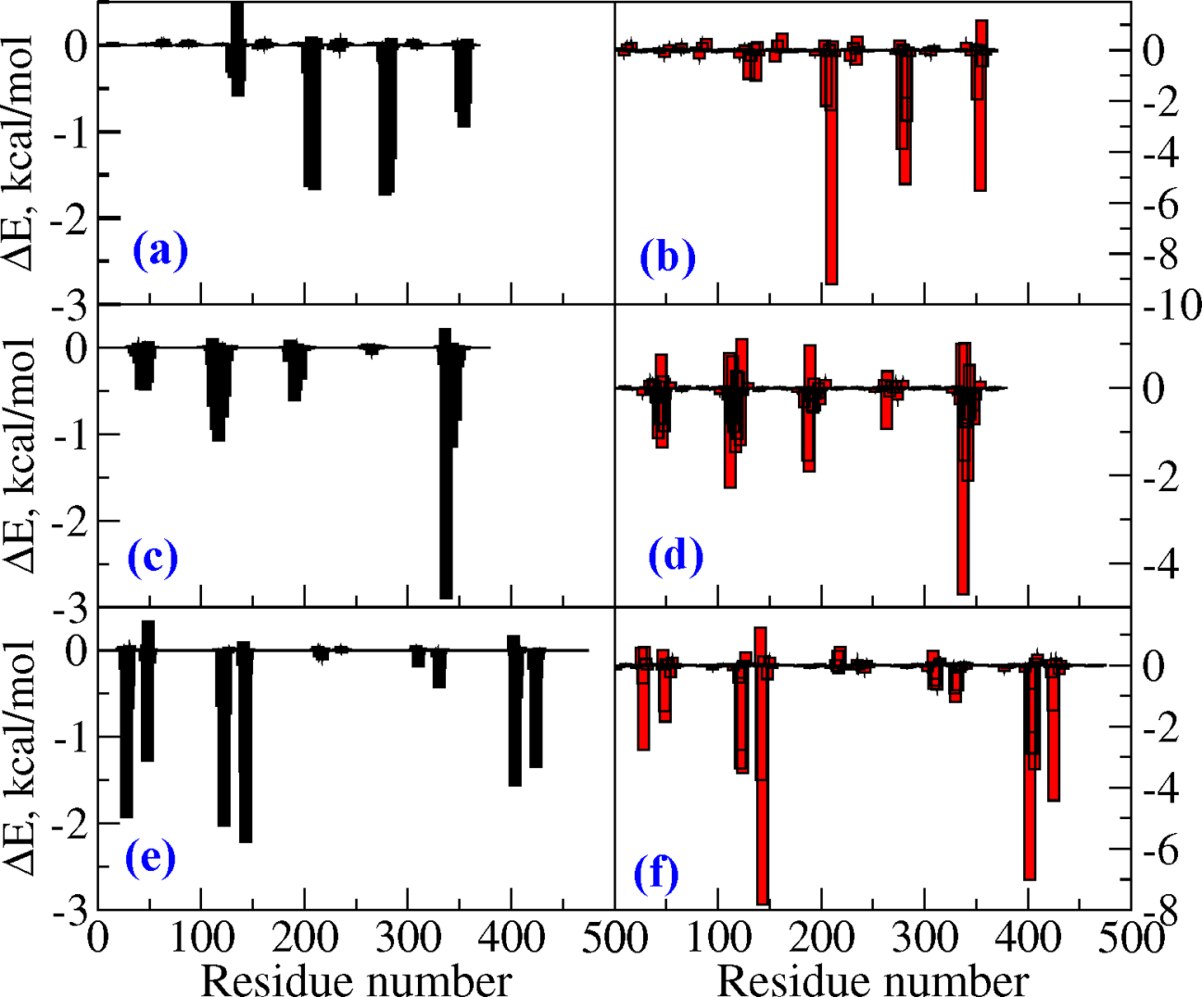
Residue-wise contributions to total binding free energy calculated from the force-field approach (subplots a, c, and e refer to AD-tau, CTE-tau, and PiD-tau, respectively) and the QM fragmentation approach (subplots b, d, and f refer to AD-tau, CTE-tau, and PiD-tau, respectively).

In order to further analyze the individual contributions to the total binding free energy, we have also plotted the residue-wise contributions, and this has been done only for the site with maximum binding affinity (refer to Figure 4). Since there are five filaments in all these fibrils, we also see a maximum of five cluster-like distributions in the profile. In the case of AD-tau, the residues PRO59, GLY62, ASN63, and LYS64 of filaments 3 and 4 are dominantly contributing to the binding free energies. They contribute as much as −1.6, −1.7, −1.4, and −1.7 kcal/mol to the total binding free energies. Except for the case of GLY62, for the remaining residues, a major part of the interactions is due to van der Waals type interactions. It is generally well established that the interaction between the amyloid and fibril-staining molecules is dominant due to hydrophobic type interactions which is as well supported in our current study. Now, let us turn to the AV-1451 tracer interaction with tau fibrils from CTE patients and let us restrict only to site 4, a core site in the fibril (as it was the one associated with maximum binding affinity). In this case only, filament 5 is contributing dominantly. The residues SER36, LEU40, and PHE42 are the ones that dominantly contribute to the total binding free energies. The contributions from these residues are, respectively, −2.9, −1.0, and −1.1 kcal/mol. The major part of the SER36 interaction with the tracer is due to electrostatic interaction, while for the other two residues, the interactions are dominantly van der Waals. This again clearly establishes that the CTE-fibril and AV-1451 association is majorly driven by hydrophobic interaction between the tracer and hydrophobic residues such as LEU40 and PHE42 and through intermolecular hydrogen bonding interaction with the SER residue. In the case of PiD-tau fibrils, the filaments 1, 2, and 5 are dominantly contributing to the total binding free energies. The residues PRO24, PRO44, and GLY45 are the dominant contributors with the individual contributions amounting to, respectively, −2.0, −1.4, and −2.2 kcal/mol, and in this case as well, the interactions between amino acids and tracer are due to hydrophobic type interactions.

In order to validate the binding free energies obtained from the MM-GBSA approach, we also carried out interaction energy calculations using the QM fragmentation scheme.^33−35^ In this case, the fibrils are cut along the peptide bonds and capped with hydrogens, and the interactions are obtained as the sum over the contributions from the individual amino acids.^39^ The total interaction energy with fibrils is referred to as *ΔE*_*fibril*_ in Table 2. The interaction energy of a tracer with solvents has also been evaluated separately and included in Table 2 as *ΔE*_*solvent*_, and this contribution mimics the solvation free energy part of the MM-GBSA scheme. The difference is that, in the current case, the tracer–solvent interaction energies are computed through the explicit solvent model, while in MM-GBSA, the solvent is treated implicitly.^32^ As can be seen, the solvation energies are very significant in the case of AD-tau and CTE-tau fibrils, while in the case of PiD-tau, the solvent contributions are relatively smaller. Further, it is striking to notice that the interaction energies between AV-1451 with different tau fibrils show the same trend as was observed from the MM-GBSA approach (refer to Table 1) which supports the reliability of the force-field-based results. The interaction energies from the QM fragmentation method show that the AV-1451 interaction with different fibrils follows the order PiD-tau > AD-tau > CTE-tau. The results from the MM-GBSA and QM fragmentation approaches are contradictory to the results from autoradiography studies with brain tissues which reported larger binding affinity toward AD-tau for the AV-1451 tracer (AD-tau > PiD-tau).^27,28^ Here, we would like to highlight that the computational modeling study carried out better mimics the in vitro binding assay study where the tracers are titrated against pure fibrils; but the experimental studies use brain homogenates as the samples, and this may contribute to a difference seen in the results from modeling and experiments. There is also a possibility that there may be certain hidden binding sites in the AD-tau fibrils which may serve as high affinity binding sites for AV-1451. This aspect will be explored in the following section.

**Table 2:** Interaction Energies of AV-1451 in Different Binding Sites of a Single Filament of Tau Fibrils from Patients with Alzheimer’s Disease, CTE, and Pick’s Disease^a^

site	*ΔE*_*fibril*_	*ΔE*_*solvent*_	*ΔE*_*total*_
site 1 of AD-tau	–35.3	–20.5	–55.8
site 4 of CTE-tau	–27.3	–22.1	–49.4
site 3 of PiD-tau	–53.1	–8.1	–61.2

a The results are obtained using the QM fragmentation scheme. The energies are in kcal/mol.

We have also carefully analyzed the residues that are largely contributing to the total interaction energies in the case of the AD-tau fibril as per the QM fragmentation scheme. The residues are PRO59, GLY62, ASN63, and LYS64, thus, the same residues that largely contributed in the case of the MM-GBSA approach. However, the interaction energies due to these residues are, respectively, −2.2, −5.3, −2.4, and −9.2 kcal/mol. This can be explained by the fact that the QM fragmentation calculations are based on the single snapshot picked up from the MD trajectory, while the MM-GBSA free energies are averaged over as many as a few hundred configurations. So, averaging of QM fragmentation-based interaction energies over various snapshots would probably yield similar results when compared to the MM-GBSA approach. Also, the interaction energies between an organic molecule and amino acid residues calculated using density functional theory and the force-field method can be quantitatively different. In the case of CTE-tau fibrils, the interaction energies due to residues SER37, LEU40, and PHE42 are, respectively, −4.7, −0.9, and −2.1 kcal/mol. Finally, in the case of PiD-tau fibrils, the contributions from the three residues namely PRO28, PRO48, and GLY49 are −3.4, −3.8, and −7.9 kcal/mol, respectively. Also in the QM fragmentation method, the residues VAL29 and ASP30 contributed significantly, and the interaction energies with the AV-1451 tracer are, respectively, −2.8 and −3.5 kcal/mol. This shows that the residues which were dominantly contributing in the MM-GBSA approach are also the ones that contributed to the interaction in the QM fragmentation-based analysis, but the energetics can be quantitatively different.

### 2.2 Identifying the Cryptic Binding Sites in AD-Derived Tau Fibrils

**Figure 5:**
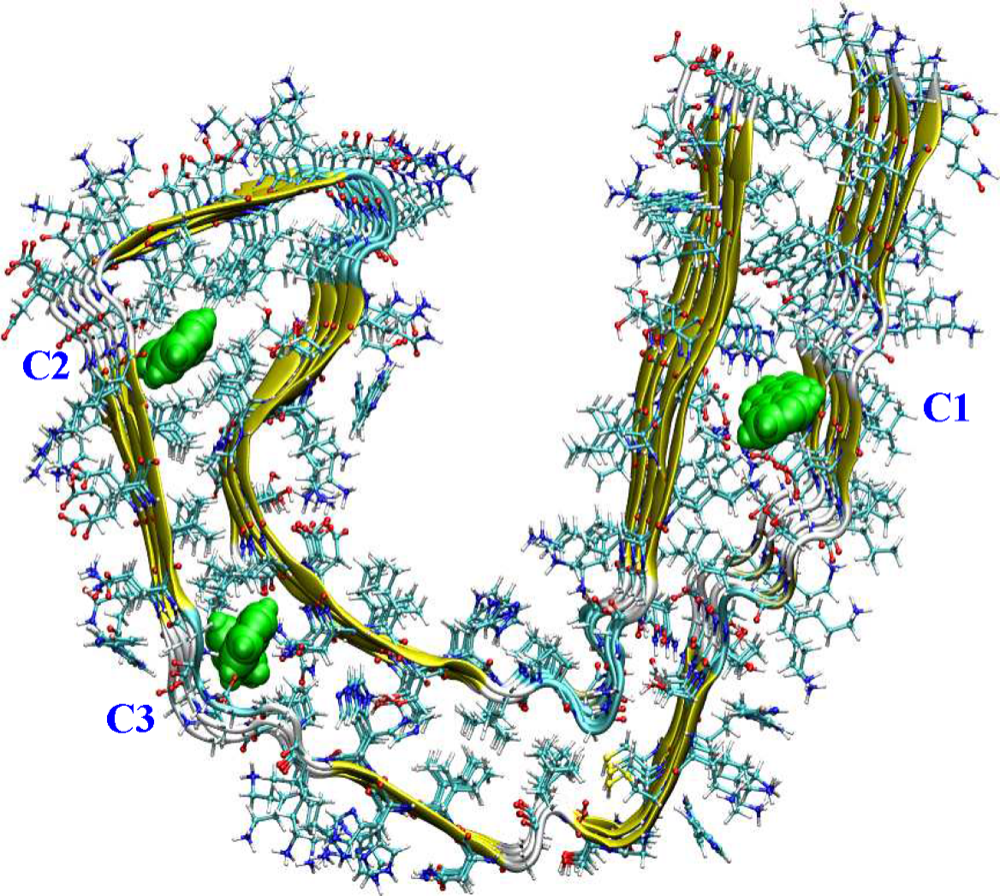
Cryptic binding sites in AD-derived tau fibrils as obtained from the organic solvent profiling approach.

**Figure 6:**
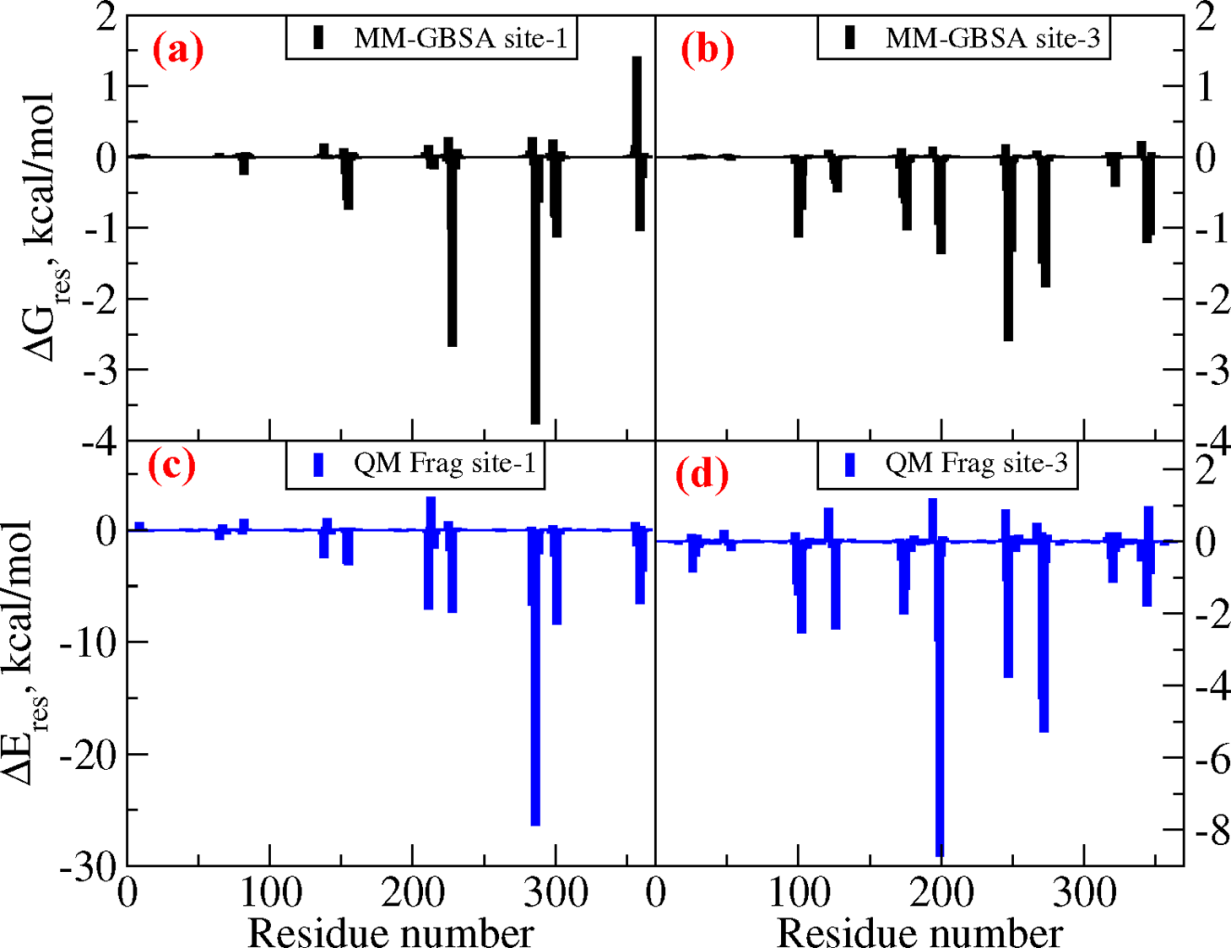
Residue-wise contributions to the total binding free energy calculated from the force-field approach (subplots a and b refer to sites 1 and 3 of AD-tau, respectively) and the QM fragmentation approach (subplots c–d refer to sites 1 and 3 of AD-tau, respectively).

The MM-GBSA- and QM fragmentation-based free energies and interaction energies suggest that the AV-1451 has larger binding affinity toward PiD-derived tau fibrils than the AD-derived tau fibrils. However, as we discussed above, the autoradiography studies with brain homogenates suggested the opposite.^27,28^ Some discrepancy can be attributed to the difference in experimental conditions and modeling studies. Further, we also wanted to check whether there are any hidden binding sites or so-called “cryptic” sites in the AD-derived tau fibrils that may have high affinity binding for the AV-1451 tracer. This speculation is based on the report of a core binding site (refer to S4 in Figure 1b) for CTE-derived tau fibrils based on the cryo-EM measurements.^11^ Even though the fragment lengths (number of amino acids in a single chain) for the tau fibrils from AD and CTE are comparable, the latter one had a core site suitable for the binding of a steroid-like hydrophobic molecule. So, we speculated that there can be certain core binding sites in AD-associated tau fibrils. Since in cyro-EM experiments the measurements are made in aqueous conditions, these sites may be hidden, but in the vicinity of small organic molecules, these sites become accessible. Interestingly, in the human genome, as many as >35% proteins are proposed to have such cryptic binding sites.^40,41^ Even the popular targets such as β-secretase and sirtuins are crystallized in closed/holo form, but when cocrystallized in the presence of inhibitors, they crystallize in apo form.^41^ The identification of such hidden sites computationally is challenging, and currently there are certain methods available: (i) long time scale molecular dynamics simulations,^42^ (ii) molecular dynamics simulations of the target in an organic solvent or mixed solvent,^43,44^ and (iii) flexible docking. In the second approach, the target biomolecule is studied in the presence of small organic solvents such as benzene, *n*-hexane, or methane or in a mixture of solvents.^43,44^ The equilibrated structure from such studies will have hidden sites exposed to facilitate the binding of organic molecules. So, by doing molecular docking, using such a fibril structure with exposed core sites will reveal additional binding for tracers which are hydrophobic in nature. In fact, a similar organic profiling study carried out for AD-derived tau fibrils in the benzene solvent and subsequent molecular docking study using a representative configuration resulted in displaying three additional core binding sites, as shown in Figure 5. We carried out molecular dynamics simulations, free energy calculations, and QM fragmentation calculations for AV-1451 bound to these three cryptic binding sites. The MM-GBSA results are presented in Table 3, and QM fragmentation-based results are given in Table 4. Site 2 among the three appears to be the one associated with minimal binding affinity. In fact, during the course of simulation, the AV-1451 bound to this site can leave easily and is localized near the entry point for this site. This observation is quite contradictory in the case of CTE-tau fibrils, where this was the site with the highest binding affinity (refer to Table 1). The striking differences in binding affinities have to be attributed to the difference in the binding site volume. It has to be optimal; and in the case of AD-tau fibrils, it is quite large, and so the tracer does not fit well. For AD-tau fibrils, the remaining two sites are high affinity binding sites. In particular, site C3 (C refers to the cryptic nature of the site) is reported to be the high affinity binding site as per the MM-GBSA approach. Again the hydrophobic interaction appears to be the dominant force in driving the complex formation. Contrary to the MM-GBSA approach, the QM fragmentation scheme predicts site C1 as the high affinity binding site for AV-1451. Independent of these differences, now the binding affinity of AV-1451 for AD-tau fibrils is larger than that for the PiD-tau fibrils. The binding affinity difference from MM-GBSA is approximately 4 kcal/mol, while the QM fragmentation scheme predicts as much as 30 kcal/mol. We have also computed the residue-wise contributions to the binding free energy for AV-1451 when it is bound to the two selected cryptic sites, and the results are displayed in Figure 6. Similarly, the residue-wise contributions to the total interaction energy computed using the QM fragmentation scheme are also shown in subplots c and d. Comparably similar patterns are observed for both cryptic sites. For example, the residues dominantly contributing continue to be similar for both MM-GBSA- and QM fragmentation-based results. However, for the case of site C1, quantitatively the contributions are very different. For example, the GLU286 contributes with 3.8 kcal/mol in the case of the MM-GBSA approach, and the stabilization is due to electrostatic contribution. In the case of the QM fragmentation scheme, this contribution amounts to −26.3 kcal/mol and is due to strong hydrogen bonding between the carboxyl group of GLU and the NH group of AV-1451. Other significant differences are observed for the residues VAL227, ASP228, and LYS357 where the quantitative estimates are different for the MM-GBSA and QM methods. Even for the case of site C3, the estimates from these two approaches are different, but the dominantly contributing residues remain the same.

**Table 3:** Binding Free Energies of AV-1451 in Different Cryptic Binding Sites of a Single Filament of Tau Fibrils from Patients with Alzheimer’s Disease^a^

site	*ΔE*_*vdw*_	*ΔE*_*elec*_	*ΔG*_*GB*_	*ΔG*_*SA*_	*ΔG*_*binding*_
site C1	–37.6	–21.0	29.2	–4.1	–33.6
site C3	–43.2	–19.4	26.2	–4.9	–41.3

a The results are obtained from the MM-GBSA approach.

**Table 4:** Interaction Energies of AV-1451 in Different Cryptic Binding Sites of Tau Fibrils from Patients with Alzheimer’s Disease^a^

site	*ΔE*_*fibril*_	*ΔE*_*solvent*_	*ΔE*_*total*_
site C1	–80.5	–14.4	–94.9
site C3	–41.1	–3.2	–44.3

a The results are obtained using the QM fragmentation scheme. The energies are in kcal/mol.

## 3 Conclusions

The objective of the present study was to gain a deeper understanding about the underlying mechanism behind the use of certain tracers to visualize different types of tauopathies, both AD and non-AD. From in vitro experiments, the tracer AV-1451 has been shown to bind to tau fibrils from AD, PSP, CBD, CTE, and other non-AD tauopathies. Studies using brain homogenates from patients with different tauopathies have reported high binding affinity for AV-1451 for the tau fibrils associated with AD when compared to fibrils associated with non-AD tauopathies.^18−20^ Here, by using an integrated computational modeling approach, we investigated in the present study the binding properties of AV-1451 to tau fibrils from AD, CTE, and PiD using the structures for these tau fibrils currently available from recent cryo-EM measurements. We employed combined molecular docking, molecular dynamics, and implicit solvent (MM-GBSA)-based free energy calculations to estimate the relative binding affinity of AV-1451 in different binding sites of the three different tau fibrils. In the cases of AD-tau and PiD-tau, both high affinity and moderate affinity binding sites were predicted, while in the case of CTE-associated tau, all the binding sites displayed comparable binding affinity. The binding affinity of AV-1451 in the core binding site of PiD-associated tau was the highest; however, when compared to surface sites, their availability for tracer binding is controlled by kinetic factors.^45^ We therefore speculate that the surface sites might be more easily available due to favorable binding kinetics than the high affinity core sites. In order to validate the force-field-based results of binding free energies, the QM fragmentation scheme was employed to compute the interaction energy between AV-1451 and different fibrils. Rewardingly, the results from the two approaches are consistent. The studies using cryo-EM structures for tau fibrils showed that the binding affinity of AV-1451 toward PiD tau fibrils was larger than that of the AD-tau fibrils which was controversial with results from autoradiography studies using brain homogenates.^27,28^ However, the cryptic sites found in AD-tau fibrils are found to be associated with high affinity for this tracer, and now the binding specificity for this tracer toward AD-fibrils is correctly reproduced as observed in experiments. The current modeling study shows that the cryo-EM structure alone may not be sufficient for certain targets, as they may have hidden high affinity binding sites. Further, the study shows that the AV-1451 association with the core site is driven mostly by hydrophobic interaction and partly by intermolecular hydrogen bonding interaction with certain polar residues. Our findings underline the possibility to design tracers that are more specific to certain types of tauopathies by optimizing interactions with local microenvironments in the core binding sites of the corresponding tau fibrils.

## 4 Methods

We have used molecular docking to find various binding sites for the AV-1451 tracer within tau protofibrils extracted from Alzheimer’s, CTE, and Pick’s disease patients. In particular, the tau fibril structures are based on cryo-EM measurements.^10,11,13^ In certain cases, the structures for paired-helical filaments are reported, and in order to be consistent in all cases, we have used the structure of a single protofibril structure as our target. Moreover, in our earlier studies, we found that the sites appearing at the interfacial region of paired helical filaments are not the ones associated with high binding affinity.^39^ Followed by molecular docking, we have carried out molecular dynamics calculations for the AV-1451 tracer when bound to various binding sites in three different tau fibrils. Since the locations of binding sites are spatially well separated from each other, we have carried out a single molecular dynamics study for each fibril–tracer complex. It would be computationally very demanding to carry out individual MD for each AV-1451 bound to different binding sites of the tau fibrils. The configurations from molecular dynamics simulations were used for the subsequent binding free energy calculations by employing the molecular mechanics-generalized Born surface area approach. In order to further validate the force-field-based binding free energies, we have also employed the QM fragmentation scheme which provides the total interaction energy between the fibril–tracer as the sum over interaction energies with various amino acid fragments. The advantage is that now the fibril interaction energies can be easily obtained at the density functional level of theory or even the MP2 level of theory. Below we elaborate on the computational details.

### 4.1 Molecular Docking Studies

The molecular structure for the tracer AV-1451 was built using the Molden software, and the geometry was optimized in the gas phase using density functional theory (in particular, B3LYP/6-31G*) by employing the Gaussian09 software.^46^ The optimized AV-1451 structure has been used as input for the molecular docking study with three different target fibrils using the Autodock4.0 software.^47^ The AD-tau fibril structure was based on the PDB structure with reference number 5O3T,^10^ while the CTE-tau and PiD-tau structures are based on the structures with PDB ids 6NWQ and 6GX5, respectively.^11,13^ The AD-tau has a pentamer unit, while the remaining two fibrils only have trimer units. So, to be consistent, we have built the pentamer protofibril structure by replicating the units along the fibril growth axis for these two cases. For the AD-tau fibrils, the number of grid points chosen in three directions for the grid box was 220 × 190 × 130. For the CTE-tau and PiD-tau, the number of grid points was chosen as 250 × 220 × 115 and 300 × 220 × 135, respectively. Since the binding sites for the tau fibrils are not known previously, the grid box dimension has been chosen to cover the whole fibril, and so, the docking software can identify both core and surface sites. The 500 low energy configurations were stored from molecular docking for further analysis. The binding poses with high binding affinity in each of the independent binding sites were chosen for subsequent molecular dynamics simulations. The high binding affinity binding sites for AV-1451 in tau fibrils from AD, CTE, and PiD patients are shown in Figure 1a–c, respectively.

### 4.2 Free Energy Calculations

The molecular dynamics simulations for complexes of AV-1451 with AD-tau, CTE-tau, and PiD-tau fibrils were carried out subsequently. As we mentioned above, the input orientations of the AV-1451 tracer within the fibrils correspond to the binding poses with the least free energy of binding from the molecular docking studies. The charges for AV-1451 were obtained by employing the CHELPG approach^48^ as implemented in Gaussian09.^46^ In this approach, the charges are obtained by best fitting to the molecular electrostatic potential. In particular, the charge calculations are performed using the B3LYP/6-31G* level of theory. In the molecular dynamics simulations, the FF99SB force field has been used to describe the fibrils. The general Amber force field and TIP3P were, respectively, used to describe AV-1451 and water molecules. The fibril–tracer complexes were solvated in the water solvent, and a sufficient number of counterions were added to neutralize the whole system. The molecular dynamics simulations were carried out using Amber 16 software.^49^ First the minimization run, followed by constant volume simulation and an equilibration simulation in the isothermal–isobaric ensemble, was carried out. The temperature was maintained at 300 K along with 1 atmospheric pressure to mimic ambient experimental conditions. The temperature and pressure were regulated by connecting the system to the Langevin thermostat and Berendsen’s barostat, respectively. The time step for the integration of equation of motion was set to be 2 fs. The time scale for the production runs was around 50 ns. During the simulation, various energetics and density properties were tested for convergence, and the simulation time scale is found to be sufficient enough. The trajectories corresponding to the last 10 ns have been used for computing the free energies of binding by employing the MM-GBSA approach. In this approach, the fibril–ligand interactions are computed by adding electrostatic and van der Waals interactions between the two subsystems. However, the polar part of solvation free energies is computed by solving the Generalized Born equation, while the nonpolar part of the solvation free energies is computed from the solvent accessible surface area. The energies are computed for all three subsystems namely AV-1451, fibril, and the fibril–AV-1451 complex, and the binding free energies are obtained as the difference between the free energy of the complex to individual systems.

### 4.3 Free Energy Calculations of AV-1451 in Cryptic Sites of AD Tau Fibrils

In order to expose the cryptic sites in AD-associated tau fibrils, this fibril has been studied in the benzene solvent. The fibril has been embedded in the benzene solvent (approximately 5000 in number), and molecular dynamics calculations involving minimization, in constant volume ensemble, and isothermal isobaric ensemble were carried out as described above in the case of the water solvent. The benzene solvent has been described using the GAFF force field, and the charges were obtained using the same approach as used for AV-1451. A short production run for a time scale of 5 ns has been carried out. The final configuration from this simulation has been used as the input configuration for molecular docking which displayed additional binding sites for AV-1451 as shown in Figure 5. A long time scale molecular dynamics for AV-1451 bound to these cryptic sites referred to as C1, C2, and C3 was carried out in the water solvent by employing the same protocol as described in the above section. Further, the binding free energy calculations for AV-1451 in the three cryptic sites (referred to as C1, C2, and C3) were computed using the same MM-GBSA approach as described above. The only difference for this set of calculations is that the starting configuration used for molecular docking is from the organic profiling study carried out for AD tau fibrils in the benzene solvent. We have also reported that the second generation tau tracer, PI2620, also binds to a cryptic site in AD-associated tau fibrils.^50^

### 4.4 QM Fragmentation Scheme for Computing the Interaction Energies

There are many reports showing the success of free energy calculation methods such as the MM-GBSA or molecular mechanics-Poisson–Boltzmann surface area (MM-PBSA) approaches. However, when the experimental binding affinity data are not available, it is recommended to compute the binding free energies with more than one computational approach to further validate the predicted results. Moreover, the relative binding affinities of tracers in different fibrils are usually very difficult to predict as the accuracy in binding free energy required for reliable prediction should be within a few kcal/mol. Since the AV-1451 binding affinity to tau fibrils from CTE and PiD is not available from experimental studies, we aimed to validate the binding free energy data with the more accurate QM fragmentation-based approach. We have developed an in-house fragmentation scheme, which can fragment the whole fibril into individual amino acids with the total fibril–ligand interaction energies computed as the sum over the fragment contributions. The fibrils are cut along the peptide bonds, and then each individual amino acid is capped either with hydrogens or with NH–CH_3_ and CO–CH_3_ groups. Also, it is possible to compute the interaction of dipeptides with the ligand so that one can also obtain the interaction energies with an account for three-body interactions. Further, the water–ligand interaction energies can be computed as well with an explicit treatment of the solvent. So, we can estimate the interactions due to the fibril alone and due to the water solvent. The interactions between ligand and fibril fragments can be computed using various levels of theory such as dispersion corrected density functional theory, MO6-2X and MP2, thus, methods known to be effective in describing the dispersion interactions. In the present study, we have computed the fibril–tracers interactions using the MO6-2X/6-31+G** level of theory. Further, the individual residue-wise contributions from each amino acid in fibrils are available, and these data have been used to validate the residue-wise decomposition of binding free energies as obtained using the MM-GBSA approach.
